# Editorial: Working anytime and anywhere: a contemporary behavioral phenomenon

**DOI:** 10.3389/fpsyg.2024.1414064

**Published:** 2024-06-19

**Authors:** Julia Schoellbauer, Clare Kelliher, Martina Hartner-Tiefenthaler

**Affiliations:** ^1^Department of Business and Psychology, Ferdinand Porsche FERNFH – Distance-Learning University of Applied Sciences, Wiener Neustadt, Austria; ^2^Cranfield School of Management, Cranfield University, Cranfield, United Kingdom; ^3^Department of Labor Science and Organization, Institute of Management Science, TU Wien, Vienna, Austria

**Keywords:** blurred boundaries, extended work, flexible work, flexible work arrangement, extended availability

Work flexibilization enables office employees and knowledge workers to work anytime and anywhere, including outside of their expected working times. In this Research Topic, we assemble seven original papers presenting theoretical considerations and empirical findings on the psychological implications of “working anytime and anywhere”. Drawing on border theory (Clark, 2000), Steffens et al. characterize the broad concept of “work-life-blending” in terms of four interlinked components: (I) the two domains of work and private life; (II) the dynamic interplay between these domains; and (III) the individual and (IV) interindividual dynamics (e.g., within a family) determining boundary segmentation strategies. The six other papers offer insights on “working anytime and anywhere” by discussing workers' *flexible work activities* (Jiang et al.; Ma et al.; Yeves et al.) and *extended work activities* (Hendrikx et al.; Mueller et al.; Schoellbauer et al.).

## Flexible working

Three papers deal with flexible working and its psychosocial implications. They frame flexible working as a resource for workers that buffers the impact of job demands and foster workers' autonomous motivation to work. More precisely, Jiang et al. reveal an increase of innovative behavior and thriving at work for remote workers, whereas Ma et al. underline their increased work engagement. Yeves et al. point toward mental health gains under work schedule flexibility due to decreased work overload, but interestingly, only for employees who are not working from home. The authors argue that working flexible hours at home may blur the boundaries between work and private life so that workers start to extend their working hours and thus, increase their workload.

## Extended working

Extended working refers to work activities or availability for work outside working hours. Behaviors such as checking work e-mails in the morning before “clocking on”, staying available for work in the evening, and finishing up work tasks on weekends extend working hours at the expense of workers' private hours and leisure time. Schoellbauer et al. systematically reviewed the vast literature on extended working and differentiate between the behavior's frequency and duration. Frequent extended working may lead to strain, whereas workers' engagement in extended working for longer durations may even have beneficial psychological implications due to learning opportunities. Nonetheless, extended working consumes time which no longer can be spent on recovery activities and can thus represent a threat to workers' health (Schoellbauer et al.). Emphasizing this risk, Hendrikx et al. showed that organizational availability norms act as a job demand that relates to workers' increased burnout symptoms due to heightened telepressure and reduced autonomy perceptions. Mueller et al. further reveal inter-individual daily fluctuations of work extending (“integration enactments”) and workers' preferences to keep their work and private life separate. They explain why workers act contra to their preferences due to work-related strain experiences (e.g., due to high workload): Strain increases preferences to separate but simultaneously cause workers to keep working to reduce the workload.

## Strength and limitations of Research Topic

The geographical variance of the collected papers (authors working in Austria, Germany, Chile, China, Netherlands, Belgium, and the UK) offers a variety of perspectives and responds to calls for more research in a wider range of national contexts. However, it should be noted that this may limit the ability for wider conclusions to be drawn since national contexts—including how the COVID-19 pandemic was experienced—may have a significant influence on the findings of individual studies. The majority of primary data presented in these contributions were collected during the COVID-19 pandemic, which may raise questions about the generalizability of the findings. Whilst in this context the physical aspects of working remotely may have been similar, the relational ones may have been different (Anderson and Kelliher, 2020). However, it is noteworthy that findings share similarities with those of earlier work as reported in the reviews of this Research Topic.

## Conclusion

Overall, the findings suggest that flexible working holds positive motivational effects as it symbolizes workers' autonomy on the job. Job autonomy is considered a resource that increases autonomous work motivation and buffers detrimental psychological effects of job stressors (Bakker and Demerouti, 2007). Despite these positive effects, flexible working might also encourage extended working which is seen as more critical. When workers engage in work anytime and anywhere, they escalate their work efforts and raise collective expectations of constant availability for work which, in turn, diminishes their job autonomy (Mazmanian et al., 2013) and jeopardizes their health (Schoellbauer et al.).

Organizations are challenged to enable flexible working while avoiding workers' self-exploitation by extending work due to a high workload. Despite the positive effects of increased work efforts, such a high performance might be only possible at the cost of workers' health. However, organizations may not want to lose those workers that are highly motivated and engaged for work even during private time. To keep those highly valued workers healthy, organizations must promote a discourse about the phenomenon of working anytime and anywhere with their workers. They must also make sure that workers regularly have periods for concentration within their working hours (e.g., focus time) to experience beneficial learning effects and enable task completion within working hours.

## Author contributions

JS: Conceptualization, Writing – review & editing, Writing – original draft. CK: Writing – review & editing, Writing – original draft, Conceptualization. MH-T: Writing – review & editing, Writing – original draft, Conceptualization.

## References

[B1] Anderson D. Kelliher C. (2020). Enforced remote working and the work-life interface during lockdown. Gender Manage.: Int. J. 35, 677–683. 10.1108/GM-07-2020-0224

[B2] Bakker A. B. Demerouti E. (2007). The job demands-resources model: State of the art. J. Manager. Psychol. 22, 309–328. 10.1108/0268394071073311531861812

[B3] Clark S. C. (2000). Work/family border theory: a new theory of work/family balance. *Hum. Relat*. 53, 747–770. 10.1177/001872670053600131275204

[B4] Mazmanian M. Orlikowski W. J. Yates J. (2013). The autonomy paradox: the implications of mobile email devices for knowledge professionals. Organizat. Sci. 24, 1337–1357. 10.1287/orsc.1120.080619642375

